# An Innovative Excited-ACS-IDGWO Algorithm for Optimal Biomedical Data Feature Selection

**DOI:** 10.1155/2020/8506365

**Published:** 2020-08-17

**Authors:** Davies Segera, Mwangi Mbuthia, Abraham Nyete

**Affiliations:** Department of Electrical and Information Engineering, University of Nairobi, Nairobi 30197, Kenya

## Abstract

Finding an optimal set of discriminative features is still a crucial but challenging task in biomedical science. The complexity of the task is intensified when any of the two scenarios arise: a highly dimensioned dataset and a small sample-sized dataset. The first scenario poses a big challenge to existing machine learning approaches since the search space for identifying the most relevant feature subset is so diverse to be explored quickly while utilizing minimal computational resources. On the other hand, the second aspect poses a challenge of too few samples to learn from. Though many hybrid metaheuristic approaches (i.e., combining multiple search algorithms) have been proposed in the literature to address these challenges with very attractive performance compared to their counterpart standard standalone metaheuristics, more superior hybrid approaches can be achieved if the individual metaheuristics within the proposed hybrid algorithms are improved prior to the hybridization. Motivated by this, we propose a new hybrid Excited- (E-) Adaptive Cuckoo Search- (ACS-) Intensification Dedicated Grey Wolf Optimization (IDGWO), i.e., EACSIDGWO. EACSIDGWO is an algorithm where the step size of ACS and the nonlinear control strategy of parameter $\vec{a}$ of the IDGWO are innovatively made adaptive via the concept of the complete voltage and current responses of a direct current (DC) excited resistor-capacitor (RC) circuit. Since the population has a higher diversity at early stages of the proposed EACSIDGWO algorithm, both the ACS and IDGWO are jointly involved in local exploitation. On the other hand, to enhance mature convergence at latter stages of the proposed algorithm, the role of ACS is switched to global exploration while the IDGWO is still left conducting the local exploitation. To prove that the proposed algorithm is superior in providing a good learning from fewer instances and an optimal feature selection from information-rich biomedical data, all these while maintaining a high classification accuracy of the data, the EACSIDGWO is employed to solve the feature selection problem. The EACSIDGWO as a feature selector is tested on six standard biomedical datasets from the University of California at Irvine (UCI) repository. The experimental results are compared with the state-of-the-art feature selection techniques, including binary ant-colony optimization (BACO), binary genetic algorithm (BGA), binary particle swarm optimization (BPSO), and extended binary cuckoo search algorithm (EBCSA). These results reveal that the EACSIDGWO has comprehensive superiority in tackling the feature selection problem, which proves the capability of the proposed algorithm in solving real-world complex problems. Furthermore, the superiority of the proposed algorithm is proved via various numerical techniques like ranking methods and statistical analysis.

## 1. Introduction

Currently, there is a growing research interest in developing and deploying population-based metaheuristics to tackle combinatorial optimization challenges. This is because they are simple, flexible with an inexpensive computational cost, and gradient-free [1].

Many researchers have applied these optimization algorithms in various research domains because of their ability to achieve best solutions.

The optimization challenge grows bigger when tackling highly dimensioned datasets. This is because these datasets have a vast feature space with many classes. Due to the presence of redundant and noninformative attributes within these datasets, the process of effective machine learning greatly hindered. Thus, the construction of efficient classifiers with high predictive power largely depends on selection of informative features [2].

Feature selection (FS) is one of the main steps in data preprocessing that aims at selecting a subset of attributes out of the whole dataset resulting into removal of noisy noninformative and redundant features. This in turn increases the accuracy of a considered classifier or clustering model [3].

FS algorithms can be broadly categorized into two classes: filter and wrapper techniques [4, 5]. Filters include techniques independent of classifiers and work directly on presented data. Moreover, these methods in many situations determine the correlations between features. On the contrary, wrapper approaches engage classifiers and mainly determine interactions between dataset features. From literature, wrapper approaches have proved to be superior compared to filters for classification algorithms [6, 7].

To utilize wrapper-based techniques, three key factors need to be outlined: considered classifiers (i.e., *k*-nearest neighbor (KNN), support vector machine (SVM)), evaluation criteria for the identified feature subset, and a search technique utilized in determining a subset of optimal features [8].

Many researchers have pointed out that determining an optimal subset of attributes is not only challenging but computationally expensive as well. Though, in the recent past, metaheuristics have proved to be reliable and efficient tools in tackling many optimization tasks (e.g., engineering designs problems, machine learning, feature selection, and data mining), they are not efficient in solving problems with high computational complexity [5, 9–11].

In the recent past, a number of metaheuristic search algorithms have been utilized for FS using highly dimensioned datasets. Some of these metaheuristics are the grey wolf optimization (GWO) [12, 13], genetic algorithm (GA) [14], particle swarm optimization (PSO) [11], ant-colony optimization (ACO) [15], differential evolution algorithm (DEA) [16], cuckoo search algorithm (CSA) [17], and dragonfly algorithm (DA) [18]. Though, many of these algorithms have already made an important contribution in the field of feature selection, in many cases, they offer acceptable solutions without a guarantee of determining optimal solutions since they do not explore the entire search space [11].

Some of the new modifications that have been proposed to improve the performance of these metaheuristics include chaotic maps [19], evolutionary methods [20], sine cosine algorithms [21], biogeography-based optimization, and local searches [22].

While designing or utilizing a metaheuristic, it should be noted that diversification (exploring the search space) and intensification (exploiting optimal solutions obtained so far) are two contradicting principles that must be balanced efficiently in order to achieve an improved performance of the metaheuristic [9].

In this regard, one promising alternative is developing a memetic algorithm whereby an integration of (at least) two algorithms is done with the aim of enhancing the overall performance.

Motivated by this, a good number of hybrid algorithms have been proposed in the recent past to solve a variety of optimizations and feature selection problems [23]. However, to enhance diversification and intensification of these hybrid algorithms, exploration and fine-tuning within their basic constituent algorithms is needed prior to hybridization [24].

This emphasizes, too, that there are a number of techniques lying within these memetic algorithms that are yet to be investigated.

Firstly, the technique of combining one or more nature-inspired algorithms (NIAs) needs to be determined. Secondly, the criterion of determining how many NIAs need to be combined within the search space has to be accomplished. Thirdly, the method of determining the application area upon which the proposed memetic algorithm has to be done. Finally, the criterion of applying the memetic algorithm in a specific domain has to be accomplished [24].

Inspired by the aforementioned, this paper proposes a new hybrid algorithm called Excited- (E-) Adaptive Cuckoo Search- (ACS-) Intensification Dedicated Grey Wolf Optimization (IDGWO), i.e., EACSIDGWO algorithm to solve the feature selection problem in biomedical science. In the proposed algorithm, the concept of the complete voltage and current responses of a direct current (DC) excited resistor capacitor (RC) circuit is innovatively utilized to make the step size of ACS and the nonlinear control strategy of parameter $\vec{a}$ of the IDGWO adaptive. Since the population has a higher diversity during early stages of the proposed algorithm, both the ACS and IDGWO are jointly utilized to attain accelerated convergence. However, to enhance mature convergence while striking an effective balance between exploitation and exploration in latter stages, the role of ACS is switched to global exploration while the IDGWO is still left conducting the local exploitation.

The remainder of this paper is organized as follows: Section 2 discusses the existing literature within the same research domain.  Section 3 presents the background information of the CS and the GWO, respectively, where their inspirations and mathematical models are given emphasis. The continuous version of the proposed EACSIDGWO algorithm is presented in Section 4 while the details of its binary version are given in Section 5. The experimental methodology considered in this paper is presented in Section 6 while the results on feature selection are discussed in Section 7. Finally, conclusions and the suggested future works are given in Section 8.

## 2. Literature Reviews

### 2.1. Review of Hybridization of GWO with Other Search Algorithms

Combining two or more metaheuristics to attain better solutions is currently a new insight in the area of optimization. In the literature, many researchers have utilized GWO in the field of hybrid metaheuristics. For instance, in [25], a hybrid of GWO and artificial bee colony (ABC) is proposed to improve performance of a complex system. In [26], GWO is hybridized with ant lion optimizer (ALO) for wrapper feature selection. Alomoush et al. [27] proposed a hybrid of GWO and harmony search (HS). In this memetic, GWO updates the bandwidth and pitch adjustment rate in HS, which in return improves the global optimization abilities of the hybrid algorithm. In [28], Arora et al. combined GWO with the crow search algorithm (CSA). The performance of the derived memetic as a feature selector is evaluated using 21 datasets. The obtained results reveal that the combined algorithm is superior in solving complex optimization algorithms. In [29], a novel combination between GWO and PSO is utilized as a load-balancing technique in the cloud-computing arena. The conclusions point out that the hybrid algorithm improved both the convergence speed and the simplicity in comparison with other algorithms. Zhu et al. [30] hybridized GWO with differential evolution (DE). The hybrid algorithm was tested on 23 different functions and a nondeterministic polynomial hard problem. The obtained results indicate that this combination achieved superior exploration. In [31], a new memetic combining the exploration ability of the fireworks algorithm (FWA) with the exploitation ability of GWO is proposed. Utilizing 16 benchmark functions with varied dimensions and complexities, the experimental results indicate that the hybrid algorithm attained attractive global search abilities and convergence speeds.

### 2.2. Review of Hybridization of CS with Other Search Algorithms

Utilizing the concept of rand and best agents within a population, Cheng et al. [32] developed an ensemble cuckoo search variant combining three different CS approaches that coexist within the entire search domain. These CS variants actively compete to derive superior generations for numerical optimization. To maintain population diversity, he introduced an external archive. The statistical results obtained reveal that the ensemble CS attained attractive converge speeds as well as robustness. In [33], GWO is hybridized with CS, i.e., GWOCS for the extraction of parameters for different PV cell models situated in different conditions. Zhang et al. [34] developed an ensemble CS algorithm that foremost divides a population into two smaller groups and then utilizes CS and differential evolution (DE) on the derived subgroups independently. The subgroups are free to share useful information by division. Further, the CS and DE algorithms can freely utilize each other's merits to complement their weaknesses. This approach proved to balance the quality of solutions and the computation consumption. In [34], CS is hybridized with a covariance matrix adaptation evolution approach, i.e., CMA-CS to improve the performance of CS in different optimization problems.

Despite the advantages portrayed by the aforementioned hybrid GWO and CS metaheuristics for optimization and feature selection, superior hybrid approaches can be achieved if the single GWO and CS algorithms are improved prior to hybridization. Furthermore, the no-free-lunch (NFL) theorem has logically proved that there has been, is, and will be no single metaheuristic capable of solving all optimization and feature selection problems [33]. While a given metaheuristic can show an attractive performance on specific datasets, its performance might degrade when applied to similar or different types of datasets [34]. Thus, there is still a dire need to improve existing algorithms or develop new ones to solve function optimization problems as well as feature selection problems efficiently.

## 3. Standard Cuckoo Search (CS)

### 3.1. Inspiration of CS

#### 3.1.1. The Behavior of Cuckoo Birds

To date, more than a thousand different species of birds are in existence in nature [35]. For most of these species, the female birds lay eggs in nests they have built themselves [36]. However, there exist some types of birds that do not build nests of their own, but instead lay their eggs in other different species' nests, leaving the responsibility of taking care of their eggs to the host birds. The cuckoos are the most famous of these brood parasites [37].

There are three types of brood parasites: intraspecific brood parasites, cooperative breeding, and nest takeover [38].The cuckoo strategy is full of amazing traits; foremost, it replaces one host egg with its own to increase the chances of its egg being hatched by the host bird. Next, it tries to mimic the pattern and color(s) of this host eggs with the aim of reducing the chances of its egg being noticed and discarded by the host bird. It is also important to point out that the timing of laying its egg is amazing since it cleverly selects a nest where a host bird has just laid eggs, implying that the cuckoo's egg will hatch prior to the host eggs. The first action taken by the hatched cuckoo is evicting the host eggs that are yet to hatch out of the nest by blind propelling in order to increase its chances of being fed well by the host bird [37]. In addition, this young cuckoo mimics the call of host chicks thus enhancing more access to the food provided by the host bird [39].

However, if this host bird is able to identify the cuckoo's egg, it can either discard it from the nest or quit this nest to build a completely new nest in a different location.

#### 3.1.2. Le'vy Flights

From literature, many researchers have shown that the behavior of many flying animals, birds, and insects can be demonstrated by a Le'vy flight [40–43]. Le'vy flights are evident when some birds, insects, and animals follow a long path with sudden turns in combination with random-short moves [43].These Le'vy flights have been successfully applied in optimization [41, 43–45]. A Le'vy flight is a random walk characterized with step lengths whose distribution is according to a heavy-tailed probability distribution.

### 3.2. Cuckoo Search (CS) Algorithm

CS is a metaheuristic swarm-based global optimization based on cuckoos that was proposed by Yang and Deb in 2009.The CS combines the obligate brood parasitic nature of cuckoos with the Le'vy flight existing in fruit flies and some birds [38]. There are three basic idealized rules for the CS, namely:

- A female cuckoo lays one egg at a time and puts it in a randomly chosen nest
- The best nests with high-quality eggs (highest fitness/solutions) will carry over to the next generations
- The number of available host nests is kept fixed, and the host bird can discover the egg laid by the female cuckoo (alien egg) with a probability *P*_*a*_ ∈ [0, 1]. Depending on the value of *P*_*a*_, the host bird can either throw away the alien egg or abandon the nest. An assumption that only a fraction of *P*_*a*_ nests are replaced by new ones

Based on the above rules, an illustration of the CS is shown in Algorithm 1.

### 3.3. Mathematical Modelling of the Standard CS

Considering Algorithm 1, the standard CS has three major steps [46–48]:

1. Exploitation (intensification) by the use of Le'vy flight random walk (LFRW)
2. Exploration (diversification) using biased selective random walk (BSRW)
3. Elitist scheme via greedy selection

#### 3.3.1. Intensification Using Le'vy Flight Random Walk (LFRW)

In this phase, new solutions are generated around the current best solution, which in return enhances the speed of the local search. This phase is achieved via the LFRW that is generally presented in (1) where the step size is derived from the Le'vy distribution.

(1) $$\begin{array}{c} X_{i,\text{gen}+1}=X_{i,\text{gen}}+\alpha ⊕\text{L}{\text{e}}^{\text{'}}\text{vy}\left(\lambda \right), \end{array}$$

where *X*_*i*,gen_ is the *i*^th^ nest in the gen^th^ generation and *X*_*i*,gen+1_ is a new nest generated by the Le'vy flight. ⊕ implies entry-wise multiplications, and *α* is the step size where *α* > 0 and is formulated in (2). The formula in equation (1) ensures that a new solution will be close to the current best solution.

(2) $$\begin{array}{c} \alpha =\alpha_{0}\times \left(X_{i,\text{gen}}-X_{\text{best}}\right), \end{array}$$

where *X*_best_ is the current solution and *α*_0_ is a scaler that is set to 0.01 in the standard CSA [38, 49].  Le′vy (*λ*) is a random number derived from the Le'vy distribution and is formulated in

(3) $$\begin{array}{c} \text{L}{\text{e}}^{\text{'}}\text{vy }\left(\lambda \right)\sim \frac{\partial \times \varepsilon }{{\left|\varphi \right|}^{1/\lambda }}, \end{array}$$

where *λ* is a constant whose value is 1.5 as suggested by Yang in the standard CS [38]. *ε* and *φ* are random numbers derived from a normal distribution whose mean and standard deviation is 1.  *∂* is a parameter computed in

(4) $$\begin{array}{c} \partial ={\left(\frac{\left\lceil \left(1+\lambda \right)\times \sin\left(\left(\pi \times \lambda \right)/2\right)\right.}{\left\lceil \left(\left(1+\lambda /2\right)\times \lambda \times 2^{\left(\left(\pi \times \lambda \right)/2\right)}\right)\right.}\right)}^{1/\lambda }, \end{array}$$

where ⌈ is a gamma function. The final form of Le'vy flight random walk (LFRW) is a combination of equations (1) to (4) as presented in

(5) $$\begin{array}{c} X_{i,\text{gen}+1}=X_{i,\text{gen}}+\alpha_{0}\;\frac{\partial \times \varepsilon }{{\left|\varphi \right|}^{1/\lambda }}\;\left(X_{i,\text{gen}}-X_{\text{best}}\right) \end{array}$$

#### 3.3.2. Diversification by the Use of Biased Selective Random Walk (BSRW)

In this phase, new solutions are randomly generated in locations far from the current best solution, an approach that ensures that the CSA is not trapped in the local optimum thus enhancing suitable diversity and exploration of the entire search space [48]. This phase of the CSA is achieved by utilizing the BSRW which is efficient in exploring the entire search space especially when it is large since the step size in the Le'vy flight is much longer in the long run [46, 48].

To find new solutions that are far from the current best solution, foremost, a trial solution is obtained by using a mutation of the current best solution and a differential step size from two solutions selected randomly. Then, a new solution is derived from a crossover operator between the current best solution and the two trial solutions [48]. The formulation of the BSRW is given in [47].

(6) $$\begin{array}{c} X_{i,\text{gen}+1}=\left\{\begin{array}{c} X_{i,\text{gen}}+s\times \left(x_{a,j,\text{gen}}-x_{b,j,\text{gen}}\right)\text{ with }P_{a}\\ X_{i,\text{gen}}\text{ with the remaining }P_{a} \end{array},\right. \end{array}$$

where *a* and *b* are two random indexes, *s* is a random number in the range [0, 1], and *P*_*a*_ is the probability discovery whose best value is 0.25 [38, 48].

#### 3.3.3. Elitist Scheme via Greedy Selection

After each random walk process, the cuckoo search algorithm utilizes the greedy strategy to select solutions with better fitness values that will be passed to the next generation. This facilitates maintenance of good solutions [48].

## 4. Grey Wolf Optimization (GWO) Algorithm

GWO is a recent nature-inspired metaheuristic algorithm that was proposed by Mirjalili et al. in 2014 [28, 50, 51]. The GWO imitates both the hunting and leadership traits of the grey wolves. The grey wolves belong to the Canidae family and follow a social hierarchy that is very strict. In most cases, a pack of between 5 and 12 wolves is involved in hunting. To efficiently simulate the leadership hierarchy of the conventional GWO algorithm, four levels are considered: alpha (*α*), beta (*β*), delta (*δ*), and omega (*ω*). Alpha, which is either a male or female is at the topmost of the hierarchy and is regarded as the leader of the pack. This leader makes all suitable decisions for the pack which are not limited to discipline and order, hunting, sleeping location, and waking-up time for the entire pack. Beta is known to assist the alpha in decision-making, and their main task is the feedback suggestions. Delta behaves like scouts, caretakers, sentinels, hunters, and elders. They control and guide the omega wolves by obeying both the beta and alpha wolves. The omega wolves are the least in the hierarchy and must obey all the other wolves [28, 50, 51].

The GWO algorithm is modelled mathematically in four stages that are described as follows.

### 4.1. Leadership Hierarchy

The mathematical model of the GWO is anchored on the social hierarchy of the grey wolves. The alpha (*α*) is considered the best solution in the population while beta (*β*) and delta (*δ*) are termed as the second and third best solutions, respectively. Lastly, the omega (*ω*) is assumed as the rest of the solutions in the population [28, 50, 51].

### 4.2. Encircling the Prey

Equation (7) and equation (8) represent the mathematical model for the wolves' encircling trait [50].

(7) $$\begin{array}{c} \vec{D}=\left|\vec{C}.{\vec{X}}_{p}\left(t\right)-\vec{X}\left(t\right)\right|, \end{array}$$

(8) $$\begin{array}{c} \vec{X}\left(t+1\right)={\vec{X}}_{p}\left(t\right)-\vec{A}.\vec{D}, \end{array}$$

where $\vec{D}$ is the distance between the prey and a given wolf. $\vec{X}$ is the wolf's position vector, and ${\vec{X}}_{p}$ depicts the prey's position vector at iteration *t*. $\vec{A}$ and $\vec{C}$ are random vectors computed as shown in [50].

(9) $$\begin{array}{c} \vec{A}=2\vec{a}.{\vec{r}}_{1}-\vec{a}, \end{array}$$

(10) $$\begin{array}{c} \vec{C}=2.{\vec{r}}_{2}, \end{array}$$

where ${\vec{r}}_{1}$ and ${\vec{r}}_{2}$ are randomly generated vectors in the range [0, 1] and $\vec{a}$ is a set vector that linearly decreases from 2 to 0 over the iterations.

### 4.3. Hunting the Prey

In the hunting stage, the alpha is considered the best applicant for the solution while its two assistants (beta and delta) are expected to know the possible location of the prey. Thus, the best three solutions that have been achieved until a given iteration are preserved and are used to compel the remaining wolves in the pack (i.e., omega) to update their positions in the search space consistent with the optimal location.

The mechanism utilized in updating the wolves' positions is given in

(11) $$\begin{array}{c} \vec{X}\left(t+1\right)=\frac{{\vec{X}}_{1}+{\vec{X}}_{2}+{\vec{X}}_{3}}{3}, \end{array}$$

where ${\vec{X}}_{1}$, $\;{\vec{X}}_{2}$, and ${\vec{X}}_{3}$ are defined and computed using

(12) $$\begin{array}{c} {\vec{X}}_{1}={\vec{X}}_{\alpha }-{\vec{A}}_{1}.{\vec{D}}_{\alpha },\\ {\vec{X}}_{2}={\vec{X}}_{\beta }-{\vec{A}}_{2}.{\vec{D}}_{\beta },\\ {\vec{X}}_{3}={\vec{X}}_{\delta }-{\vec{A}}_{3}.{\vec{D}}_{\delta }, \end{array}$$

where ${\vec{X}}_{\alpha }$, ${\vec{X}}_{\beta }$, and ${\vec{X}}_{\delta }$ are the three best wolves (solutions) in the pack at a given iteration *t*. ${\vec{A}}_{1}$, ${\vec{A}}_{2}$, and ${\vec{A}}_{3}$ are calculated using Equation (9), while $\;{\vec{D}}_{\alpha }$, ${\vec{D}}_{\beta }$, and ${\vec{D}}_{\delta }$ are calculated using

(13) $$\begin{array}{c} {\vec{D}}_{\alpha }=\left|{\vec{C}}_{1}.{\vec{X}}_{\alpha }-\vec{X}\right|,\\ {\vec{D}}_{\beta }=\left|{\vec{C}}_{2}.{\vec{X}}_{\beta }-\vec{X}\right|,\\ {\vec{D}}_{\delta }=\left|{\vec{C}}_{3}.{\vec{X}}_{\delta }-\vec{X}\right|, \end{array}$$

where ${\vec{C}}_{1}$, $\;{\vec{C}}_{2},$ and ${\vec{C}}_{3}$ are calculated based on Equation (10).

### 4.4. Searching and Attacking the Prey

The grey wolves can only attack the prey when it stops moving. This is modelled mathematically based on vector $\vec{A}$ that is utilized in Equation (9). Vector $\vec{A}$ is comprised of values that span within the range [−2*a*, 2*a*], and the value of $\vec{a}$ is decreased from 2 to 0 over the course of iterations using

(14) $$\begin{array}{c} \vec{a}=2-\left(\frac{2\times \text{iter}}{{\mathrm{Max}}_{\text{iter}}}\right), \end{array}$$

where iter is the iteration number and Max_iter_ is the optimal total number of iterations.

When $\left|\vec{A}\right|<1$, the wolves are forced to attack the prey, and when $\left|\vec{A}\right|>1$, the wolf diverges out from the current prey.Searching for the prey is the exploration phase while attacking it is the exploitation phase.

## 5. Excited-Adaptive Cuckoo Search-Intensification Dedicated Grey Wolf Optimization (EACSIDGWO)

In general, effective balancing between diversification (global search) and intensification (local search) in a metaheuristic plays a beneficial and crucial role in achieving excellent performance of an algorithm [52–54]. However, it is difficult to achieve this balance with a single metaheuristic (for example, either using CSA or GWO) [52, 53]. For instance, CSA is efficient at exploring the promising area of the whole search space (diversification) but ineffective at fine-tuning the end of the search space (exploitation/intensification) [55, 56]. On the other hand, GWO is good at intensification (exploitation) but inefficient at diversification (exploration) [32, 57].

For this reason, in trying to enhance mature convergence while ensuring that the required effective balance between diversification and intensification is met, a hybrid algorithm called Excited-Adaptive Cuckoo Search-Intensification Dedicated Grey Wolf Optimization (EACSIDGWO) utilizing the strengths of each algorithm (i.e., CSA's diversification and GWO's intensification abilities) is proposed in this paper. Moreover, the adaptability of the proposed EACSIDGWO is guided innovatively by the complete voltage and current responses of a DC excited RC circuit (whose analysis results in first order differential equations) that finds continual applications in electronics, communications, and control systems [58].

### 5.1. Adaptive Cuckoo Search (ACS)

#### 5.1.1. Adaptive Step Size via the Complete Voltage Response of the DC Excited RC Circuit

From the details of the standard CS algorithm presented in Section 2, it is evident that the algorithm lacks a criterion to control its step size through the iteration process. Control of the step size is key in guiding the CS algorithm to reach either its global maxima or minima [48, 59].

Inspired by the complete voltage response of a direct current (DC) excited RC circuit which increases with time, a novel mechanism to control the step size is proposed. Contrary to prior research [48, 59] where the step size decays with generations, in this research, the step size grows with generations with the aim of strengthening the diversification (exploration) ability of the CS, which is a component of the proposed EACSIDGWO algorithm.

The solution to the first order differential equation of the direct current-excited RC circuit motivated the formulation of a new variant of ACS in this paper.

The complete voltage response of the RC circuit to a sudden application of a DC voltage source, with the assumption that the capacitor is initially not charged, is given in

(15) $$\begin{array}{c} v\left(t\right)=\left\{\begin{array}{cc} 0, & t<0,\\ V_{s}\left(1-e^{-\tau /\tau }\right), & t>0, \end{array}\right. \end{array}$$

where *τ* = *R*∗*C* is the time constant, which expresses the rapidity with which this voltage *v*(*t*) rises to the value of *V*_*s*_ which is a constant DC voltage source. *R* and *C* are the equivalent resistance and capacitance in the circuit, respectively.

Considering the situation when *t* > 0, equation (15) can be rewritten as presented in

(16) $$\begin{array}{c} v\left(t\right)=V_{s}\left(1-{\left(e^{-t}\right)}^{\tau }\right),\\ v\left(t\right)=V_{s}\left(1-{\left(\frac{1}{e^{t}}\right)}^{\tau }\right). \end{array}$$

As *t* → ∞, the component (1/*e*^*t*^) → 0 forcing *v*(*t*→∞) → *V*_*s*_. We adopt this concept, i.e., the exponential growth of *v*(*t*) to control the step size of the cuckoo search algorithm by introducing the proposed

(17) $$\begin{array}{c} {\text{step}}_{\text{gen}+1}={\text{step}}_{\mathrm{Max}}\times \left(1-{\left(\frac{{\text{gen}}_{\mathrm{Max}}-\text{gen}}{{\text{gen}}_{\mathrm{Max}}}\right)}^{\tau }\right), \end{array}$$

where gen is the current generation (iteration), step_Max_ is the upper bound of the step size step, and gen_Max_ is the maximum number of generations (iterations).

To ensure that the step_gen+1_ is proportional to the fitness of a given individual nest within the search space in the current generation, the nonlinear modulation index *τ* is formulated in

(18) $$\begin{array}{c} \tau_{i,\text{gen}}=\left|\frac{\left(\left({\alpha_{\text{nest}f}}_{\text{gen}}+{\beta_{\text{nest}f}}_{\text{gen}}+{\delta_{\text{nest}f}}_{\text{gen}}\right)/3\right)-{{\text{i}}_{\text{nest}f}}_{\text{gen}}}{\left(\left(\left({\alpha_{\text{nest}f}}_{\text{gen}}+{\beta_{\text{nest}f}}_{\text{gen}}+{\delta_{\text{nest}f}}_{\text{gen}}\right)/3\right)\right)-{{\text{worst}}_{\text{nest}f}}_{\text{gen}}}\right|, \end{array}$$

where *τ*_*i*,gen_ is the nonlinear modulation index for the *i*^th^ nest in generation gen, *α*_nest*f*__gen_ is the fitness value of the alpha (*α*) nest (overall best nest) in generation gen,  *β*_nest*f*__gen_ is the fitness value of the beta (*β*) nest (2^nd^ best nest) in generation gen, *δ*_nest*f*__gen_ is the fitness value of the delta (*δ*) nest (3^rd^ best nest) in generation gen, i_nest*f*__gen_ is the fitness value of the *i*^th^ nest in generation gen, and worst_nest*f*__gen_ is the fitness value of the worst nest among the remaining omega (*ω*) nests (i.e., nests whose fitness values are not featured among the top three fitness values).

Thus, equation (17) is further modified as

(19) $$\begin{array}{c} {\text{step}}_{i,\text{gen}+1}={\text{step}}_{\mathrm{Max}}\times \left(1-{\left(\frac{{\text{gen}}_{\mathrm{Max}}-\text{gen}}{{\text{gen}}_{\mathrm{Max}}}\right)}^{\tau_{i,\text{gen}}}\right), \end{array}$$

where step_*i*,gen+1_ is the step size for the for the *i*^th^ nest in generation gen + 1. From equation (19), the step size step_*i*,gen+1_ is nonlinearly increasing from relatively small values to values close to step_Max_. The reason for proposing a nonlinearly increasing strategy are as follows. Foremost, at the early stages of the proposed EACSIDGWO algorithm, whereby ACS is a component, the population has a higher diversity. A higher diversity implies a stronger ability to explore the global space. Our aim at this point is to accelerate convergence. Therefore, the value of the step size step_*i*,, gen+1_ is set to a smaller value.

It is important to point out that the anticipated accelerated convergence is a joint effort attained by foremost setting the step_*i*,gen+1_ of the ACS to a small value at early stages and utilizing the IDGWO (whose details are presented in Section 4.2) whose core task is exploitation.

On the other hand, since the proposed EACSIDGWO algorithm is a hybrid algorithm where the ACS cooperatively works with the IDGWO, all the nests will be attracted to the global optima, i.e., the alpha (*α*) nest at the later stage. This will compel them to converge prematurely without being given enough room to explore the search space. Such a situation will lead the nests away from a local optimum and encourage diversification. For this reason, the value of the step size step_*i*,gen+1_ is set to a larger value, i.e., step_Max_. In this paper, the step_Max_ is set to 1.

In other words, our main reason for proposing a nonlinearly increasing step size step_*i*, gen+1_ is that its small values at the initial stages of the proposed EACSIDGWO algorithm facilitates “local exploitation” while its larger values in the later stages will facilitate “global exploration”.

The ACS can then be modeled as presented in

(20) $$\begin{array}{c} X_{i,\text{gen}+1}=X_{i,\text{gen}}+\mathrm{rand}n\times {\text{step}}_{i,\text{ gen}+1}. \end{array}$$

Equation (20) is a formulation of the new search space for the ACS from the current solution.

Moreover, if this step size is considered proportional to the global best solution, then equation (20) can be formulated as given in

(21) $$\begin{array}{c} X_{i,\text{gen}+1}=X_{i,\text{gen}}+\mathrm{rand}n\times {\text{step}}_{i,\text{ gen}+1}*\left(X_{i,\text{gen}}-X_{g\text{best},\text{ gen}}\right), \end{array}$$

where *X*_*g*best,gen_ is the global best solution among all *X*_*i*_ for *i* = 1, 2, ⋯, *n* at generation gen, and *n* is the number of host bird nests.

Thus, from equations (17), (18), (19), (20), (21), it is evident that the diversification ability of the ACS is heightened as the number of generations (gen) approach the maximum number of generations (gen_Max_). This is because the value of the step size rapidly increases towards the set maximum value of step (step_Max_).

### 5.2. Intensification Dedicated Grey Wolf Optimizer (IDGWO)

#### 5.2.1. Nonlinearly Controlling Parameter $\vec{a}$ via the Complete Current Response of the DC Excited RC Circuit

It is evident from Section 4.4 that parameter $\vec{a}$ plays a critical role in balancing the diversification (exploration) and the intensification (exploitation) of a search agent.

A large value of control parameter $\vec{a}$ facilitates diversification while a smaller value of this parameter facilitates intensification. Thus, a suitable selection of the control parameter $\vec{a}$ can enhance a good balance between global diversification (exploration) and local intensification (exploitation).

In the original GWO (described in Section 3), the value of $\vec{a}$ linearly decreases from 2 to 0 (refer to equation (14)). However, the search process of the GWO algorithm is both nonlinear and complicated, which cannot be truly reflected by the linear control strategy of $\vec{a}$ presented in equation (14).

In addition, Mittal et al. [60] proposed that an attractive performance can be attained if parameter $\vec{a}$ is nonlinearly decreased rather than decreased linearly.

Inspired by the complete current response of a direct current (DC) excited RC circuit which increases with time, a novel nonlinear adjustment mechanism of control parameter $\vec{a}$ is formulated in this paper.

The complete current response of the RC circuit to a sudden application of a DC voltage source, with the assumption that the capacitor is initially not charged, is given in

(22) $$\begin{array}{c} i\left(t\right)=\frac{V_{s}}{R}\left({\left(\frac{1}{e^{t}}\right)}^{\tau }\right). \end{array}$$

As *t* → ∞, the component (1/*e*^*t*^) → 0 forcing *i*(*t*→∞) → 0. We adopt this concept, i.e., the exponential decay of *i*(*t*) to formulate a novel improved strategy, i.e., equation (23) to generate the values for control parameter$\;\vec{a}$.

(23) $$\begin{array}{c} {\vec{a}}_{i,\text{gen}}=a_{o}\times {\left(\frac{{\text{gen}}_{\mathrm{Max}}-\text{gen}}{{\text{gen}}_{\mathrm{Max}}}\right)}^{\tau_{i,\text{gen}}}, \end{array}$$

where gen is the current generation (iteration), *a*_*o*_ is the initial higher value of parameter  *a* and gen_Max_ is the maximum number of generations (iterations).  *τ*_*i*,gen_ is the nonlinear modulation index described earlier by equation (18).

Consequently, vector $\vec{A}$ is computed as given in

(24) $$\begin{array}{c} \vec{A}=2{\vec{a}}_{i,\text{gen}}.{\vec{r}}_{1}-{\vec{a}}_{i,\text{gen}}. \end{array}$$

Equation (23) is a nonlinear decreasing control parameter for $\;{\vec{a}}_{i,\text{gen}}$ whose initial upper limit is equal to the value *a*_*o*_ while its final lower limit is zero.

From the original literature of GWO, the value $\left|\vec{A}\right|<1$ compels the grey wolves to move towards the prey (exploitation) while $\left|\vec{A}\right|>1$ compels them to move away from the prey in search of a fitter prey (exploration). Thus, setting *a*_*o*_ to 1 will always force the wolves to move to the prey which will enable us the dedicated modified GWO algorithm, a component of proposed EACSIDGWO, for intensification.

#### 5.2.2. Enhanced Mature Convergence via a Fitness Value-Based Position-Updating Criterion

Both diversification and intensification are crucial for population-based optimization algorithms [60]. However, from the detailed account of the conventional GWO (refer to Section 3), it is evident that all the other wolves are attracted towards the three leaders *α*, *β*, and *δ*; a scenario that will force the algorithm to converge prematurely without attaining sufficient diversification of the search space. In other words, the conventional GWO is prone to premature convergence.

In reference to the position-updated criterion of GWO described by equation (11), a new candidate individual is obtained by moving the old individual towards the best leader (*α* wolf), the second best leader (*β* wolf), and the third best leader (*δ* wolf). This approach will force all the other grey wolves to crowd in a reduced section of the search space that might be different from the optimal region and without giving them a leeway to escape from such a region. In an effort to overcome this major drawback, in this paper, a scheme that promotes mature converge is devised.

Instead of averaging the values of vectors $\;{\vec{X}}_{1}$, ${\vec{X}}_{2}$, and ${\vec{X}}_{3}$ (a form of recombining them) as a mechanism of updating the wolves' positions (refer to equation (11)), in this paper, we make full use of the information of their fitness values as a criteria of arriving at new positions for the wolves.

Foremost, the search agents of the populations ${\vec{X}}_{1}$, ${\vec{X}}_{2}$, and ${\vec{X}}_{3}$ are computed as given in

(25) $$\begin{array}{c} {\vec{X}}_{1}\left(i,j\right)={\vec{X}}_{\alpha }\left(j\right)-{\vec{A}}_{1}.{\vec{D}}_{\alpha }, \end{array}$$

(26) $$\begin{array}{c} {\vec{X}}_{2}\left(i,j\right)={\vec{X}}_{\beta }\left(j\right)-{\vec{A}}_{2}.{\vec{D}}_{\beta }, \end{array}$$

(27) $$\begin{array}{c} {\vec{X}}_{3}\left(i,j\right)={\vec{X}}_{\delta }\left(j\right)-{\vec{A}}_{3}.{\vec{D}}_{\delta }, \end{array}$$

where *i* = 1, 2, ⋯, *n* and *j* = 1, 2, ⋯, *d*. *n* is the population size while *d* is the dimension of the search space.

Next, the fitness value for each search agent in each of the derived populations, i.e., ${\vec{X}}_{1}$,$\;{\vec{X}}_{2},$ and ${\vec{X}}_{3}$ is evaluated. Further, a new population with the fittest values is derived from these three populations, i.e., ${\vec{X}}_{1}$,$\;{\vec{X}}_{2},$ and ${\vec{X}}_{3}$.

Equations (28) and (29) represent the process undertaken to derive this new population.

(28) $$\begin{array}{c} =\max\left(\overset{3}{\underset{j=1}{⋃}}X_{j}f_{i,\text{gen}}\right), \end{array}$$

(29) $$\begin{array}{c} X_{i,\text{gen}+1}=\overset{3}{\underset{j=1}{⋃}}{{\vec{X}}_{j_{i,\text{gen}}}}_{\text{Index}}, \end{array}$$

where ${\vec{X}}_{j_{i,\text{gen}}}$ is vector *j* computed using search agent *i* during iteration  gen, *X*_*j*_*f*_*i*,gen_ is the fitness value of vector ${\vec{X}}_{j_{i,\text{gen}}}$.

### 5.3. Proposed EACSIDGWO (Continuous Version)

We cooperatively combined the proposed adaptive cuckoo search (ACS) and the intensification-dedicated grey wolf optimization (IDGWO) and developed the EACSIDGWO. In the EACSIDGWO algorithm, the ACS is actively involved in intensification (exploitation) during the early stage when the population has higher diversity and diversification at later stages. On the other hand, the IDGWO is only actively involved in intensification in all the stages of the proposed algorithm. By doing so, an effective balance between diversification and intensification is achieved. In addition, mature convergence is enhanced which in the end leads to high-quality solutions.

## 6. Proposed EACSIDGWO (Binary Version)

Selection of features is binary by nature [61]. Therefore, the proposed EACSIDGWO algorithm cannot be utilized in selection of features without further modifications.

In the proposed EACSIDGWO algorithm, the new positions of the search agents will have continuous solutions, which must be converted into corresponding binary values.

In this paper, this conversion is achieved by foremost applying squashing of the continuous solutions in each dimension using a sigmoid (S-shaped) transfer function [61]. This will compel the search agents to move into a binary search space as depicted by equation (31).

(30) $$\begin{array}{c} S=\frac{1}{1+e^{-10\left({X^{d}}_{i,\text{gen}}-0.5\right)}}, \end{array}$$

where *X*^*d*^_*i*,gen_ is a continuous-valued position of the *i*^th^ search agent in the *d*^th^ dimension during generation gen.

The output *S* of the sigmoid transfer function is still a continuous value, and thus, it has to be the threshold to reach the binary-value one. Normally, the sigmoid function maps smoothly the infinite input to a finite output [61]. To arrive at the binary solution when a sigmoid function is used, the commonly stochastic threshold is applied as presented in

(31) $$\begin{array}{c} {y^{d}}_{i,\text{gen}}=\left\{\begin{array}{c} 0\text{ if }\mathrm{rand}<S,\\ 1\text{ if }\mathrm{rand}\geq S, \end{array}\right. \end{array}$$

(32) $$\begin{array}{c} Y_{i,\text{gen}}=\overset{n}{\underset{i=1}{⋃}}{y^{d}}_{i,\text{gen}}, \end{array}$$

where *y*^*d*^_*i*,gen_ is the binary updated position at generation gen in the *d*^th^ dimension and rand is a random number drawn from a uniform distribution ∈[0, 1]. *Y*_*i*,gen_ is the equivalent binary vector of the *i*^th^ search agent at generation gen.

Using this approach, the original solutions remain in the continuous domain of the proposed EACSIDGWO algorithm and can be converted to binary when the need arises.

The pseudocode of the binary version of the proposed EACSIDGWO algorithm is presented in Algorithm 3.

## 7. Experimental Methodology

In this section, detailed accounts of the biomedical datasets, evaluation metrics, proposed fitness function, and the parameter setting for the considered metaheuristic algorithms are outlined.

### 7.1. Considered Biomedical Datasets

To validate the performance of the considered metaheuristic algorithms, six benchmark biomedical datasets extracted from the UCI Irvine Machine [62] were utilized. Each dataset has two classes, and the performance of each of these algorithms is evaluated based on its ability to classify these classes correctly. Details of these datasets are given in Table 1.

### 7.2. Evaluation Metrics

For the considered feature selection problem, the following evaluation metrics were utilized to compare the performance of each considered feature selection technique.

*Average Accuracy (Avg_Acc).* It is one of the commonly used classification metric that represents the number of correctly classified instances by using a particular feature set. The mathematical formulation of this metric is given in Equation (33).

(33) $$\begin{array}{c} \text{AvglowbarAcc}=\frac{1}{N}\overset{N}{\underset{i=1}{\sum }}\frac{1}{k}\overset{k}{\underset{j=1}{\sum }}{\text{Acc}}_{j}, \end{array}$$

where *N* is the number of times (runs) a given metaheuristic algorithm is run, *k* represents the number of folds utilized, and Acc_*j*_ is the accuracy reported during fold *j*. Acc_*j*_ is defined in equation (34).

(34) $$\begin{array}{c} {\text{Acc}}_{j}=\frac{{\text{TP}}_{j}+{\text{TN}}_{j}}{{\text{TP}}_{j}+{\text{TN}}_{j}+{\text{FP}}_{j}+{\text{FN}}_{j}}, \end{array}$$

where TP and FN denote the number of positive samples in fold *j* that are accurately and falsely predicted, respectively, and TN and FP represent the number of negative samples in the same fold that are predicted accurately and wrongly, respectively [63].

*Average Feature Length (Avg_NFeat).* This metric characterizes the average length of selected features to the total number of features in the dataset. Equation (35) gives its mathematical formulation.

(35) $$\begin{array}{c} \text{AvglowbarNFeat}=\frac{1}{N}\overset{N}{\underset{i=1}{\sum }}\text{S}\text{ellowbar}{\text{Feat}}_{i}, \end{array}$$

where SellowbarFeat_*i*_ is the number of selected features in the testing dataset during run *i*.

*Minimum Accuracy (Min_Acc).* It is the least value of accuracy reported during *N* runs. Equation (36) depicts its formulation.

(36) $$\begin{array}{c} \mathrm{Min}\text{lowbarAcc}=\min\left(\overset{N}{\underset{j=1}{⋃}}\text{A}\text{vglowbarcrossAc}{\text{c}}_{j}\right), \end{array}$$

where AvglowbarcrossAcc_*i*_ is given by

(37) $$\begin{array}{c} \text{AvglowbarcrossAc}{\text{c}}_{i}=\frac{1}{k}\overset{k}{\underset{j=1}{\sum }}{\text{Acc}}_{j}. \end{array}$$

*Maximum Accuracy (Max_Acc)*. It is the largest value of accuracy reported during *N* runs. Its mathematical formulation is given by

(38) $$\begin{array}{c} \mathrm{Max}\text{lowbarAcc}=\max\left(\overset{N}{\underset{j=1}{⋃}}{{\text{Avg}}_{\text{crossAcc}}}_{j}\right). \end{array}$$

*Maximum Features Selected (Max_NFeat).*It is the largest number of selected features during *N* runs. Equation (39) gives its mathematical formulation.

(39) $$\begin{array}{c} \mathrm{Max}\text{lowbarNFeat}=\max\left(\overset{N}{\underset{i=1}{⋃}}{{\text{Sel}}_{\text{Feat}}}_{i}\right). \end{array}$$

*Minimum Features Selected (Min_NFeat).* It is the least number of selected features during *N* runs. Equation (40) gives its mathematical formulation.

(40) $$\begin{array}{c} \mathrm{Min}\text{lowbarNFeat}=\min\left(\overset{N}{\underset{i=1}{⋃}}{{\text{Sel}}_{\text{Feat}}}_{i}\right). \end{array}$$

### 7.3. Evaluation of the Classifier Performance

Since the support vector machine classifier has already made immense contributions in the field of microarray-based cancer classification [63], it was adopted in this paper to evaluate the classification accuracy using the selected subset of features returned by the various considered metaheuristic feature selection approaches. The Matlab fitcsvm function that trains and cross-validates an SVM model was adopted in this paper. We specified the kernel scale parameter to “auto” to allow the function to select the appropriate scale factor using a heuristic search.

With the SVM classifier, the data items are mapped points in an *n*−dimensional feature space (i.e., *n* = number of features) and each feature's value is a value of a given coordinate. The final output of this classifier is an optimal hyperplane which can be used to classify new cases [17, 63].

However, the performance of the SVM classifier is highly dependent on the selection of its kernel function [17, 63]. A reason why experiments were conducted using various kernels in this paper.

Selecting a suitable kernel is both dataset and problem specific and selected experimentally [17, 63]. Based on the conducted experiments, suitable kernel functions were selected for the considered datasets. The considered datasets and their suitable kernel functions are presented in Table 2.

More information of selecting suitable SVM kernel functions is presented in [63].

### 7.4. Fitness Function

The main aim of a feature selection exercise is to discover a subset of features from the whole set of existing features in a given dataset such that the considered optimization algorithm is able to achieve the highest possible accuracy using that subset. For instance, in datasets with many features (attributes), the objective is to minimize the number of selected features while improving the classification accuracy of the feature selection approach.

In classifications tasks, there exist higher chances that two feature subsets containing a different number of features will have the same accuracy [17]. However, if a subset with a large number of features is discovered earlier by a given optimization algorithm, it is likely that the one with least features will be ignored [17].

In trying to overcome this challenge, a fitness function proposed in [17] to evaluate the classification performance of optimization algorithms for feature selection tasks is adopted. This fitness function is given in

(41) $$\begin{array}{c} \text{Fit}=\alpha *\frac{\left|R\right|}{\left|N\right|}-\beta *\text{AvglowbarcrossAc}{\text{c}}_{i}, \end{array}$$

where |*N*| represents the total number of features within a given dataset, |*R*| represents the number of selected features during run *i*, and AvglowbarcrossAcc_*i*_ is the average cross-validation accuracy reported during run *i* (refer to Equation (37)). *β* and *α* are two weights corresponding to the significance of the classification quality and the subset length, respectively. In this paper, *β* is set to 0.8 and *α* = 0.2 as adopted from [17].

It is important to point out that both terms are normalized by division by their largest possible values; i.e., the number of selected features |*R*| is divided by the total number of features |*N*|, and average accuracy AvglowbarcrossAcc_*i*_ is divided by the value 1.

### 7.5. Parameter Setting for the Considered Feature Selection Techniques

The performance of the proposed EACSIDGWO algorithm was compared to those of extended binary cuckoo search (EBCS), binary ant-colony optimization (BACO), binary genetic algorithm (BGA), and binary particle swarm optimization (BPSO) that were reported earlier in [17].

Table 3 indicates the selected parameter values for both the proposed BEACSIDGWO algorithm and each of the other algorithms as reported in [17].

To be consistent with the setup proposed in [17], the population size for the proposed EACSIDGWO was set to 30. Then, the algorithm was run 10 times to perform the feature selection task for each considered dataset. In addition, each run terminated when 10000 fitness function evaluations was attained. This approach allowed the proposed algorithm to utilize the fitness function at an equal number of times.

In this paper, all the experiments were conducted using Matlab 2017 running on Windows 10 operating system on a HP desktop with Intel® Core™ i7-3770CPU @ 3.4 GHZ with 12.0 GB of RAM.

## 8. Results and Discussion

To examine the diversification and intensification of the proposed EACSIDGWOA, detailed comparative study is presented in this section.

The efficiency and the optimization performance of the proposed algorithm have been verified by comparing and analyzing its results with those of four other state-of-the-art optimization algorithms.

The experimental classification results have been probed through statistical tests, comparative analysis, and ranking methods.

Tables 4–9 provide the performance of all the considered optimization approaches for feature selection using the datasets described in Section 7.1. It is important to point out that the best result achieved in each column for all the considered biomedical datasets is highlighted in bold while the worst is italicized.

To prove that the proposed EACSIDGWO is superior over the other four-optimization algorithms, Wilcoxon rank-sum test, i.e., a nonparametric statistical test, is also performed. The statistical results for the *p*, *h*, and *z* values obtained from the pairwise comparisons of the four groups are tabulated in Table 10. Tables 11 and 12 present a comparison of the overall ranking of the results obtained by the considered algorithms.

### 8.1. Discussion

#### 8.1.1. Investigation of the Obtained Classification Results

From Tables 4–9, the following observations can be made.

- The proposed EACSIDGWO algorithm outperformed all the other considered algorithms in terms of classification accuracy for all the utilized datasets. It recorded the highest classification accuracy on the three highly dimensioned datasets (i.e., Ovarian, CNS, and Colon) as well as the remaining three small sample-sized datasets. This promising performance is largely attributed to the cooperative exploitation conducted by ACS and IDGWO components of the proposed algorithm during the early generations, as well as the single-handed exploitation and exploration by IDGWO and ACS, respectively, at later generations
- For four datasets, i.e., Ovarian, Heart, CNS and Colon, the proposed algorithm attained a value for  AvglowbarAcc that is larger than the value for MaxlowbarAcc attained by the EBCS. EBCS is a variant of cuckoo search, which is a component of the proposed EACSIDGWO algorithm. This superior performance proves the competency of the proposed approach to efficiently determine the optima within the search space
- With regard to the average feature length (AvglowbarNFeat), the proposed B-EACSIDGWO algorithm demonstrated a superior performance by selecting the least number of features compared to the other algorithms. According to the results reported in Tables 4–9, the proposed algorithm performed better on all the considered datasets

In comparison with the original number of features in the considered datasets, there is a notable reduction in the number selected features by the proposed approach. For instance, the actual number of features in ovarian cancer, CNS, and Colon cancer datasets is 4000, 7129, and 2000, respectively, whereas the number of selected features by the proposed EACSIDGWO is 274.8, 1208.1, and 538.5, respectively. This clearly indicates that the proposed algorithm is able to reduce the number of features as well as locate the most significant optimal feature subsets. The strength of the proposed EACSIDGWO lies in its well-formulated algorithm (refer to Section 5) that enhances both its diversification and intensification capabilities which enables it to eliminate redundant (noninformative) attributes and then actively searches within the high-performance regions of the feature space.

#### 8.1.2. Statistical Analysis

The superiority of the proposed EACSIDGWO algorithm has been verified via Wilcoxon rank-sum test, i.e., a nonparametric test with a significance level of 5%. The results obtained for the pairwise comparison of the four groups are presented in Table 10. Observations from Table 10 reveal the statistical significance of the obtained experimental results for all the considered datasets. This clearly indicates that the proposed approach has an attractive performance in relation to the other four approaches. Thus, the overall statistical results by our algorithm are highly significant from the results of the four algorithms for all the considered datasets.

#### 8.1.3. Ranking Methods

Tables 11 and 12 outline the detailed ranking of all the considered algorithms with their respective comparative analysis. The ranking is based on maximum accuracy (MaxlowbarAcc), minimum accuracy (MinlowbarAcc), average accuracy (AvglowbarAcc), maximum number of selected features (MaxlowbarNFeat), minimum number of selected features (MinlowbarNFeat), and average number of selected features (AvglowbarNFeat). From the ranking, it is evident that the proposed EACSIDGWO algorithm obtained the best values in all these measures for all the datasets. Considering the final ranks, the proposed algorithm attained an attractive performance whose overall rank value is 37.This clearly reveals the superiority of EACSIDGWO algorithm in relation to the four state-of-the-art algorithms.

## 9. Conclusion

This paper proposed a new hybrid Excited- (E-) Adaptive Cuckoo Search- (ACS-) Intensification Dedicated Grey Wolf Optimizer (IDGWO), i.e., EACSIDGWO algorithm to solve the feature selection problem in biomedical science. In the proposed algorithm, the concept of the complete voltage and current responses of a direct current (DC) excited resistor capacitor (RC) circuit are innovatively utilized to make the step size of ACS and the nonlinear control strategy of parameter $\vec{a}$ of the IDGWO adaptive. Since the population has a higher diversity during early stages of the proposed algorithm, both the ACS and IDGWO are jointly utilized to attain accelerated convergence. However, to enhance mature convergence while striking an effective balance between exploitation and exploration in later stages, the role of ACS is switched to global exploration while the IDGWO is still left conducting the local exploitation. In order to test the efficiency of the proposed EACSIDGWO as a feature selector, six standard biomedical datasets from the University of California at Irvine (UCI) repository were utilized. The experimental results obtained prove that the proposed algorithm is superior to the state-of-the-art feature selection techniques, i.e., BACO, BGA, BPSO, and EBCSA in attaining a good learning from fewer instances and optimal feature selection from information-rich biomedical data, all these while maintaining a high classification accuracy of the utilized data. In the future, utilizing this hybrid algorithm as a filter-feature selection approach seeking to evaluate the generality of the selected features will be a valuable contribution.

## Figures and Tables

**Algorithm 1:**
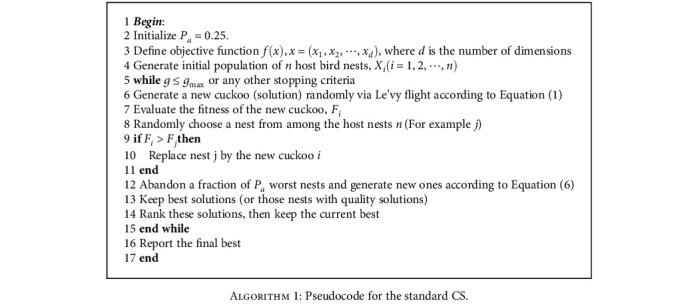
Algorithm 1: Pseudocode for the standard CS.

**Algorithm 2:**
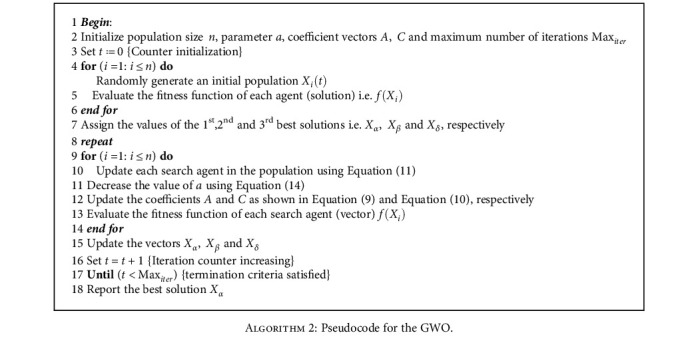
Algorithm 2: Pseudocode for the GWO.

**Algorithm 3:**
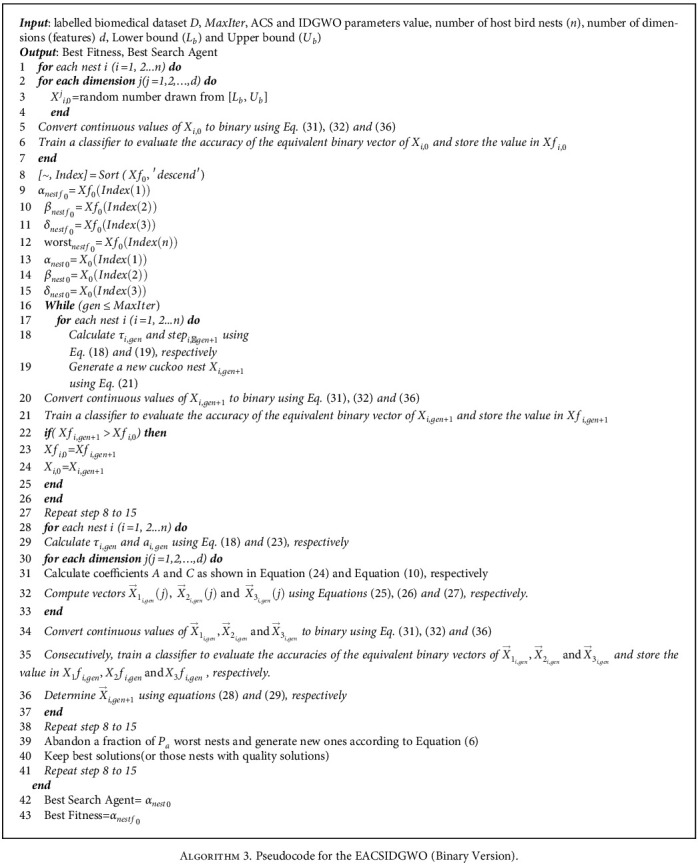
Algorithm 3. Pseudocode for the EACSIDGWO (Binary Version).

**Table 1:** Considered biomedical datasets.

Dataset	Number of features	Number of cases
Breast Cancer Wisconsin (prognosis)	33	198
Breast Cancer Wisconsin (diagnostic)	30	569
SPECTF Heart	44	267
Ovarian Cancer	4000	216
CNS	7129	60
Colon	2000	62

**Table 2:** Selection of suitable kernel functions.

Dataset	Kernel function
Breast Cancer Wisconsin (prognosis)	Radial basis function (RBF)
Breast Cancer Wisconsin (diagnostic)	Radial basis function (RBF)
SPECTF Heart	Radial basis function (RBF)
Ovarian Cancer	Linear function
CNS	Linear function
Colon	Linear function

**Table 3:** Selection of parameter values for the considered approaches.

Algorithm	Parameter values
EACSIDGWO	step_Max_ = 1, *a*_*o*_ = 1, and *P*_*a*_ =0.25
EBCS	*N*_mut_ = 10, *λ* = 1, *α* = 1, and *P*_*a*_=0.4
BACO	Γ_initial_ = 0.1, *α* = 1, and *p* = 0.1
BGA	*M*_*r*_ = 0.1, *C*_*r*_ = 0.1
BPSO	*C*_1_ = 1, *C*_2_ = 2, *ω*_initial_ = 0.9, *ω*_vary−for_ = 0.9

**Table 4:** Experimental results for the Ovarian Cancer dataset.

Algorithm	Accuracy			Number of features		
	MaxlowbarAcc	MinlowbarAcc	AvglowbarAcc	MaxlowbarNFeat	MinlowbarNFeat	AvglowbarNFeat
EACSIDGWO	**1.000**	**1.000**	**1.000**	**292**	**264**	**274.8**
EBCS	*0.991*	0.991	0.991	1855	1747	1811.6
BACO	*0.991*	*0.986*	*0.990*	*1971*	*1912*	*1945.7*
BGA	*0.991*	0.991	0.991	1830	1755	1887.3
BPSO	*0.991*	*0.986*	*0.990*	1913	1777	1857

Values in bold represent the best result, and values in italic denote the worst in each column, respectively.

**Table 5:** Experimental results for the Breast Cancer Wisconsin (Diagnostic) dataset.

Algorithm	Accuracy			Number of features		
	MaxlowbarAcc	MinlowbarAcc	AvglowbarAcc	MaxlowbarNFeat	MaxlowbarNFeat	AvglowbarNFeat
EACSIDGWO	0.977	**0.974**	**0.975**	**3**	**3**	**3**
EBCS	**0.981**	**0.974**	0.973	4	**3**	3.1
BACO	*0.972*	*0.960*	*0.969*	*8*	*6*	*7*
BGA	0.975	0.965	0.972	6	**3**	3.6
BPSO	**0.981**	0.963	0.974	*8*	**3**	5.4

Values in bold represent the best result, and values in italic denote the worst in each column, respectively.

**Table 6:** Experimental results for the Breast Cancer Wisconsin (Prognosis) dataset.

Algorithm	Accuracy			Number of features		
	MaxlowbarAcc	MinlowbarAcc	AvglowbarAcc	MaxlowbarNFeat	MinlowbarNFeat	AvglowbarNFeat
EACSIDGWO	**0.879**	**0.864**	**0.873**	**7**	**3**	**5.6**
EBCS	0.874	0.828	0.856	8	4	6.2
BACO	*0.818*	*0.768*	*0.794*	*12*	*5*	*8.4*
BGA	0.874	0.793	0.843	10	4	6.5
BPSO	*0.848*	*0.798*	0.821	11	4	8.3

Values in bold represent the best result, and values in italic denote the worst in each column, respectively.

**Table 7:** Experimental results for the SPECTF Heart dataset.

Algorithm	Accuracy			Number of features		
	MaxlowbarAcc	MinlowbarAcc	AvglowbarAcc	MaxlowbarNFeat	MinlowbarNFeat	AvglowbarNFeat
EACSIDGWO	**0.884**	**0.861**	**0.875**	**6**	**3**	**4.5**
EBCS	0.873	0.846	0.861	8	5	6.2
BACO	*0.846*	*0.813*	*0.831*	*15*	*10*	*12.1*
BGA	**0.884**	0.846	0.866	11	4	8.4
BPSO	0.865	0.846	0.854	*15*	9	10.9

Values in bold represent the best result, and values in italic denote the worst in each column, respectively.

**Table 8:** Experimental results for the CNS dataset.

Algorithm	Accuracy			Number of features		
	MaxlowbarAcc	MinlowbarAcc	AvglowbarAcc	MaxlowbarNFeat	MinlowbarNFeat	AvglowbarNFeat
EACSIDGWO	**0.767**	**0.700**	**0.718**	**1623**	**807**	**1208.1**
EBCS	*0.667*	0.667	0.667	3490	3391	3446.7
BACO	*0.667*	*0.650*	*0.660*	*3589*	3432	*3522.9*
BGA	0.683	0.667	0.668	3566	*3438*	3489.7
BPSO	*0.667*	0.667	0.667	3547	3359	3474.3

Values in bold represent the best result, and values in italic denote the worst in each column, respectively.

**Table 9:** Experimental results for the Colon dataset.

Algorithm	Accuracy			Number of features		
	MaxlowbarAcc	MinlowbarAcc	AvglowbarAcc	MaxlowbarNFeat	MinlowbarNFeat	AvglowbarNFeat
EACSIDGWO	**0.919**	**0.887**	**0.905**	**637**	**397**	**538.5**
EBCS	0.903	0.871	0.887	*1016*	*961*	*988.7*
BACO	0.903	0.871	*0.881*	1002	932	976
BGA	*0.887*	0.871	0.882	1003	944	962.8
BPSO	*0.887*	*0.855*	0.879	1003	933	971.2

Values in bold represent the best result, and values in italic denote the worst in each column, respectively.

**Table 10:** Using Wilcoxon's rank-sum test at *p* = 0.05 to compare EACSIDGWO with other algorithms.

Dataset	Wilcoxon's rank-sum test	EBCS vs EACSIDGWO	BACO vs EACSIDGWO	BGA vs EACSIDGWO	BPSO vs EACSIDGWO
Ovarian Cancer	*p* value	0.000181651	0.000181651	0.000182672	0.000181651
	*h* value	1.000000000	1.000000000	1.000000000	1.000000000
	*z* value	3.743255786	3.743255786	3.741848283	3.743255786

Breast Cancer Wisconsin (diagnostic)	*p* value	0.022591996	0.000146767	0.017044126	0.000582314
	*h* value	1.000000000	1.000000000	1.000000000	1.000000000
	*z* value	2.28026466	3.796476695	2.38575448	3.439721266

Breast Cancer Wisconsin (prognosis)	*p* value	0.000730466	0.0001707	0.00073729	0.000174624
	*h* value	1.000000000	1.0000000	1.00000000	1.000000000
	*z* value	3.377881495	3.758843896	3.375323463	3.753152986

SPECTF Heart	*p* value	0.000321376	0.000176611	0.000176611	0.000177611
	*h* value	1.000000000	1.000000000	1.000000000	1.000000000
	*z* value	3.597430949	3.750317207	3.750317207	3.748901726

CNS	*p* value	0.000182672	0.000182672	0.000182672	0.000182672
	*h* value	1.000000000	1.000000000	1.000000000	1.000000000
	*z* value	3.741848283	3.741848283	3.741848283	3.741848283

COLON	*p* value	0.000182672	0.000182672	0.000182672	0.000181651
	*h* value	1.000000000	1.000000000	1.000000000	1.000000000
	*z* value	3.741848283	3.741848283	3.741848283	3.743255786

**Table 11:** Overall ranking of considered algorithms.

Algorithm	Measures	Datasets									
		Ovarian Cancer	Breast Cancer Wisconsin (diagnostic)	Breast Cancer Wisconsin (prognosis)	SPECTF Heart	CNS	Colon	Sum of ranks	Overall rank	Total sum	Final ranks
EACSIDGWO	MaxlowbarAcc	1	2	1	1	1	1	7	1	37	1
	MinlowbarAcc	1	1	1	1	1	1	6	1		
	AvglowbarAcc	1	1	1	1	1	1	6	1		
	MaxlowbarNFeat	1	1	1	1	1	1	6	1		
	MinlowbarNFeat	1	1	1	1	1	1	6	1		
	AvglowbarNFeat	1	1	1	1	1	1	6	1		

EBCS	MaxlowbarAcc	2	1	2	3	3	2	13	2	84	2
	MinlowbarAcc	2	1	2	2	2	2	11	2		
	AvglowbarAcc	2	2	2	3	3	2	14	2		
	MaxlowbarNFeat	3	2	2	2	2	4	15	2		
	MinlowbarNFeat	2	1	2	3	3	5	16	2		
	AvglowbarNFeat	2	2	2	2	2	5	15	2		

BACO	MaxlowbarAcc	2	4	4	4	2	2	18	4	138	5
	MinlowbarAcc	3	4	5	3	3	2	20	4		
	AvglowbarAcc	3	5	5	5	4	4	26	5		
	MaxlowbarNFeat	5	4	5	4	5	2	25	5		
	MinlowbarNFeat	5	2	3	5	3	2	20	3		
	AvglowbarNFeat	5	5	5	5	5	4	29	5		

**Table 12:** Overall ranking of considered algorithms.

Algorithm	Measures	Datasets									
		Ovarian Cancer	Breast Cancer Wisconsin (diagnostic)	Breast Cancer Wisconsin (prognosis)	SPECTF Heart	CNS	Colon	Sum of ranks	Overall rank	Total sum	Final ranks
BGA	MaxlowbarAcc	2	3	2	1	2	3	13	2	95	3
	MinlowbarAcc	2	2	5	2	2	2	15	3		
	AvglowbarAcc	2	4	3	2	2	3	16	3		
	MaxlowbarNFeat	2	3	3	3	4	3	18	3		
	MinlowbarNFeat	3	1	2	2	4	4	16	2		
	AvglowbarNFeat	3	3	3	3	3	2	17	3		

BPSO	MaxlowbarAcc	2	1	3	3	3	3	15	3	110	4
	MinlowbarAcc	3	3	2	2	2	3	15	3		
	AvglowbarAcc	3	2	4	4	3	5	21	4		
	MaxlowbarNFeat	4	4	4	4	3	3	22	4		
	MinlowbarNFeat	4	1	2	4	2	3	16	2		
	AvglowbarNFeat	3	4	4	4	3	3	21	4		

## Data Availability

The data used to support the findings of this study are available from the corresponding author upon request.
